# The Effects of Cobalt Protoporphyrin IX and Tricarbonyldichlororuthenium (II) Dimer Treatments and Its Interaction with Nitric Oxide in the Locus Coeruleus of Mice with Peripheral Inflammation

**DOI:** 10.3390/ijms20092211

**Published:** 2019-05-05

**Authors:** Patricia Moreno, Rafael Alves Cazuza, Joyce Mendes-Gomes, Andrés Felipe Díaz, Sara Polo, Sergi Leánez, Christie Ramos Andrade Leite-Panissi, Olga Pol

**Affiliations:** 1 Grup de Neurofarmacologia Molecular, Institut d’Investigació Biomèdica Sant Pau, Hospital de la Santa Creu i Sant Pau, 08025 Barcelona, Spain; patricia.morenov@e-campus.uab.cat (P.M.); andresfelipe.diaz@e-campus.uab.cat (A.F.D.); sara.polor@e-campus.uab.cat (S.P.); sergi.leanez@uab.es (S.L.); 2 Institut de Neurociències, Universitat Autònoma de Barcelona, 08193 Barcelona, Spain; 3 Department of Psychology, Faculty of Philosophy, Science and Letters, University of São Paulo, Ribeirão Preto 14040-901, SP, Brazil; cazuzarafaelalves@usp.br (R.A.C.); joypharm1@yahoo.com.br (J.M.-G.); christie@usp.br (C.R.A.L.-P.)

**Keywords:** analgesia, carbon monoxide, heme oxygenase 1, inflammatory pain, locus coeruleus, nitric oxide

## Abstract

Heme oxygenase 1 (HO-1) and carbon monoxide were shown to normalize oxidative stress and inflammatory reactions induced by neuropathic pain in the central nervous system, but their effects in the locus coeruleus (LC) of animals with peripheral inflammation and their interaction with nitric oxide are unknown. In wild-type (WT) and knockout mice for neuronal (NOS1-KO) or inducible (NOS2-KO) nitric oxide synthases with inflammatory pain induced by complete Freund’s adjuvant (CFA), we assessed: (1) antinociceptive actions of cobalt protoporphyrin IX (CoPP), an HO-1 inducer; (2) effects of CoPP and tricarbonyldichlororuthenium(II) dimer (CORM-2), a carbon monoxide-liberating compound, on the expression of HO-1, NOS1, NOS2, CD11b/c, GFAP, and mitogen-activated protein kinases (MAPK) in the LC. CoPP reduced inflammatory pain in different time-dependent manners in WT and KO mice. Peripheral inflammation activated astroglia in the LC of all genotypes and increased the levels of NOS1 and phosphorylated extracellular signal-regulated kinase 1/2 (p-ERK 1/2) in WT mice. CoPP and CORM-2 enhanced HO-1 and inhibited astroglial activation in all genotypes. Both treatments blocked NOS1 overexpression, and CoPP normalized ERK 1/2 activation. This study reveals an interaction between HO-1 and NOS1/NOS2 during peripheral inflammation and shows that CoPP and CORM-2 improved HO-1 expression and modulated the inflammatory and/or plasticity changes caused by peripheral inflammation in the LC.

## 1. Introduction

The effects of carbon monoxide and nitric oxide on the regulation of the nociceptive responses induced by acute painful stimuli, chronic inflammation, or nerve injury and those associated with diabetic neuropathy have been investigated [1,2,3,4], but the possible interaction among them has been poorly evaluated.

Several studies demonstrated that, whereas carbon monoxide is a potent modulator of inflammation and nociception, nitric oxide has a more complex role in the development and maintenance of chronic pain. That is, while treatment with carbon monoxide inhaled and/or released by tricarbonyldichlororuthenium(II)dimer (CORM-2) exerted robust antiinflammatory [5,6] and antinociceptive actions during inflammatory and neuropathic pain [2,7,8], both types of pain were inhibited with the administration of selective nitric oxide synthase (NOS) inhibitors or in mice lacking neuronal (NOS1-KO) or inducible (NOS2-KO) nitric oxide synthases [9,10,11,12,13].

More interesting is the detail that, although the interaction among carbon monoxide and nitric oxide has been widely investigated at the vascular level [14,15,16], in the control of fewer, sepsis, hemorrhagic shock, etc. [17,18], only few studies examined this interaction in pain regulation. In this line, previous works revealed that carbon monoxide required the NOS pathway for its antinociceptive effects, whereas nitric oxide effects were produced independently of the carbon monoxide system [19,20]. Nonetheless, the interaction among heme oxygenase 1 (HO-1) enzyme, principally responsible for the antinociceptive effects induced by carbon monoxide [2], and NOS1 or NOS2 in inflammatory pain has not been evaluated. In this study, we assessed this interaction by testing the antinociceptive effects of cobalt protoporphyrin IX (CoPP), an HO-1 inducer, in both NOS1- and NOS2-deficient mice with chronic peripheral inflammation.

Recent works revealed that the systemic administration of CoPP and CORM-2, besides inhibiting neuropathic pain and blocking NOS1 and NOS2 over-expression in the spinal cords [2,21], activated powerful anti-inflammatory and antioxidant responses in several brain areas [22]. Considering that the locus coeruleus (LC) is implicated in the control of nociception [23] and both HO-1 [24,25] and NOS1/NOS2 enzymes [26,27] are well expressed in it, our objective was to evaluate the potential interaction between them in the LC of animals with inflammatory pain. 

Several authors have demonstrated the relevant role played by glial cells in the development and maintenance of pain [28]. Therefore, and considering the inhibitory effects induced by CoPP and CORM-2 treatments on glial activation induced by sciatic nerve injury in the spinal cord and specific brain areas such as amygdala and hippocampus [2,22], in this study, we also evaluated the effects of these treatments on the expression of GFAP (an astroglial marker) and CD11b/c (a microglial marker) in LC of animals with complete Freund’s adjuvant (CFA)-induced inflammatory pain. 

It is well known that peripheral inflammation, in addition to induce the phosphorylation of several mitogen-activated protein kinases (MAPK)—especially, the extracellular signal-regulated kinase 1/2 (ERK 1/2) and c-Jun N-terminal kinase (JNK) in the spinal cord [29,30]—also activated ERK 1/2 in LC [31,32]. Considering that the systemic and peripheral administration of CoPP and other antiinflammatory agents, such as diclofenac, normalized the up regulation of p-ERK 1/2 and p-JNK induced by chronic pain in different brain areas [22,32], we examined the effects of CoPP and CORM-2 on the expression of p-ERK 1/2 and p-JNK in LC of animals with inflammatory pain.

Then, using wild-type (WT), NOS1-KO, and NOS2-KO mice with chronic peripheral inflammation, we assessed the antinociceptive actions of the repeated administration of CoPP and the effects of CoPP and CORM-2 treatments on the protein levels of HO-1, NOS1, NOS2, CD11b/c, GFAP, p-ERK ½, and p-JNK in the LC of these animals.

## 2. Results

### 2.1. Antinociceptive Effects of CoPP in WT, NOS1-KO, and NOS2-KO Mice

**Figure 1:**
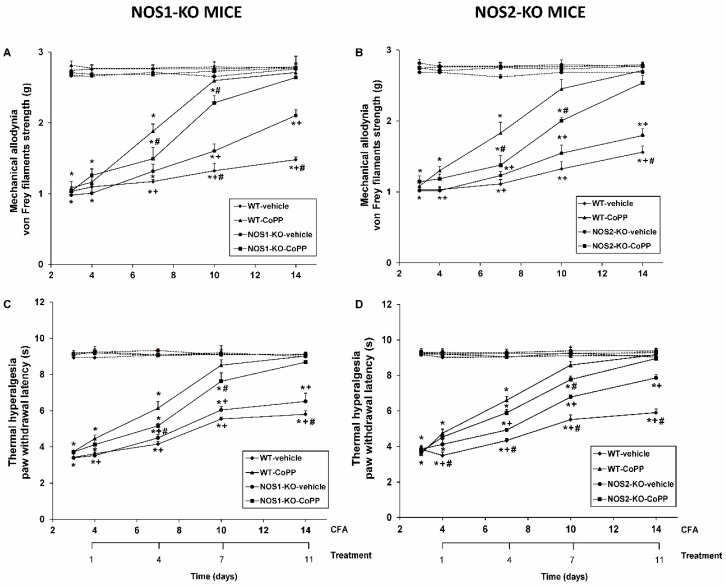
Antiallodynic and antihyperalgesic effects of cobalt protoporphyrin IX (CoPP) in wild-type (WT), neuronal nitric oxide synthase knock-out (NOS1-KO), and inducible nitric oxide synthase knock-out (NOS2-KO) mice with peripheral inflammation. Mechanical allodynia (**A**,**B**) and thermal hyperalgesia (**C**,**D**) in ipsilateral (continuous lines) and contralateral paws (discontinuous lines) of WT, NOS1-KO, and NOS2-KO mice treated for 11 days with vehicle or CoPP at 4, 7, 10, and 14 days after complete Freund’s adjuvant (CFA) injection are shown. For each genotype, day and treatment were assessed; * denotes significant differences when compared with their respective contralateral paw, + denotes significant differences when compared with their respective ipsilateral paw treated with CoPP, and # denotes significant differences of the same treatment between genotypes (*p* < 0.05, one-way ANOVA, Student–Newman–Keuls test). Data are shown as mean values ± SEM; *n* = 8 animals per group.

Mechanical allodynia and thermal hyperalgesia were assessed after 1, 4, 7, and 11 days of repeated administration of CoPP or vehicle in WT, NOS1-KO, and NOS2-KO mice (Figure 1). 

Our data showed that CFA injection caused mechanical allodynia in the ipsilateral paw of all genotypes from day 3 to day 14 since CFA injection (*p* < 0.001; one-way ANOVA vs. their respective contralateral paws). In NOS1-KO animals (Figure 1A), three-way ANOVA displayed significant effects of the genotype on day 11 (*p* < 0.011), of the treatment on days 4, 7, and 11 (*p* < 0.020), and with respect to the paw on days 0, 1, 4, 7, and 11 of CoPP treatment (*p* < 0.001). In addition, significant interactions among genotype and treatment at days 7 and 11 (*p* < 0.042), genotype and paw at day 14 (*p* < 0.005), treatment and paw at days 4, 7, and 11 (*p* < 0.017), and genotype, treatment, and paw at days 7 and 11 of CoPP treatment (*p* < 0.019) were also demonstrated. Our results, besides confirming that NOS1-KO mice had faster recovery of the mechanical allodynia than WT animals from days 10 to 14 after CFA injection (*p* < 0.001, one-way ANOVA), demonstrated that mechanical allodynia caused by CFA was further reduced in WT compared to NOS1-KO mice after 4 and 7 days of CoPP treatment (*p* < 0.001, one-way ANOVA; Figure 1A).

In NOS2-KO animals (Figure 1B), three-way ANOVA also showed effects of treatment at days 1, 4, 7, and 11 (*p* < 0.007), and with respect to the paw at days 0, 1, 4, 7, and 11 of CoPP administration (*p* < 0.001). Moreover, significant interactions amongst genotype and treatment at day 7 (*p* < 0.007), treatment and paw at days 4, 7, and 11 (*p* < 0.025), as well as among genotype, treatment, and paw at days 4, 7, and 11 days of CoPP treatment (*p* < 0.032) were evident. Therefore, although similar mechanical allodynia caused by CFA was observed in WT and NOS2-KO animals, the inhibitory effects of CoPP were stronger in WT that in NOS2-KO mice at 4 and 7 days of treatment (*p* < 0.001, one-way ANOVA, Figure 1B). In all genotypes, no treatment produced no effect on the respective contralateral paw.

In all genotypes, peripheral inflammation also reduced the threshold for evoking ipsilateral paw withdrawal to thermal stimulus from days 3 to 14 after CFA injection (*p* < 0.001; one-way ANOVA vs. the corresponding contralateral paw) (Figure 1C,D). 

In NOS1-KO animals, three-way ANOVA proved significant effects of treatment at days 1, 4, 7, and 11 (*p* < 0.002) and of paw at days 0, 1, 4, 7, and 11 of CoPP administration (*p* < 0.001). Moreover, significant interactions among genotype and treatment at day 4 (*p* < 0.008), treatment and paw at days 1, 4, 7, and 11 of CoPP treatment (*p* < 0.018), and among genotype, treatment, and paw at days 4 and 7 of CoPP treatment in NOS1-KO mice were demonstrated (*p* < 0.045) (Figure 1C). Therefore, although similar thermal hyperalgesia induced by CFA was observed in both genotypes, its inhibition by CoPP was higher in WT mice than in NOS1-KO animals at days 4 and 7 of treatment (*p* < 0.001, one-way ANOVA).

In NOS2-KO mice, our data confirmed that the absence of this isoform improved the recovery from thermal hyperalgesia induced by CFA (Figure 1D). In addition, significant effects of genotype at days 7 and 11 (*p* < 0.010), treatment at days 1, 4, 7, and 11 (*p* < 0.004), and paw at days 0, 1, 4, 7, and 11 of CoPP treatment (*p* < 0.001) were demonstrated. Moreover, significant interactions among genotype and treatment at days 4, 7, and 11 (*p* < 0.010), treatment and paw on days 1, 4, 7, and 11 (*p* < 0.005), as well as among genotype, treatment, and paw at days 4, 7, and 11 of CoPP administration (*p* < 0.002) were shown. Thus, the reduced thermal hyperalgesia induced by CoPP in WT mice was similar to that induced by this compound in NOS2-KO animals during the overall experiment, except at day 7 of treatment, in which it was higher in WT than in NOS2-KO mice (*p* < 0.05; one-way ANOVA). In all genotypes, the absence of treatment did not produced any effect on the respective contralateral paw.

### 2.2. Effects of CoPP and CORM-2 on HO-1, NOS1, NOS2, CD11b/c, GFAP, p-ERK 1/2, and p-JNK Expression in the LC of WT Mice with Peripheral Inflammation

**Figure 2:**
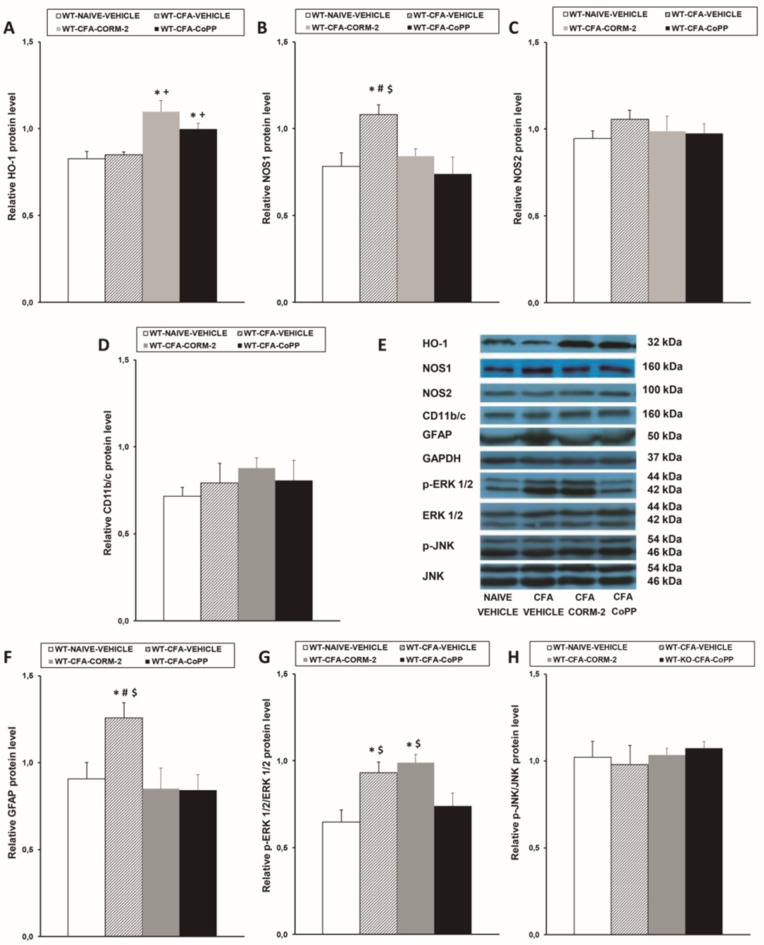
Effects of CoPP and tricarbonyldichlororuthenium(II) dimer (CORM-2) on the protein levels of HO-1, NOS1, NOS2, CD11b/c, GFAP, p-ERK 1/2, and p-JNK in the LC of WT mice. Protein levels of HO-1 (**A**), NOS1 (**B**), NOS2 (**C**), CD11b/c (**D**), GFAP (**F**), p-ERK 1/2 (**G**), and p-JNK (**H**) in the LC from CFA-injected WT mice treated with vehicle, CORM-2, or CoPP are represented. These levels in naïve mice treated with vehicle are also represented as controls. In all panels, * denotes significant differences vs. naïve vehicle-treated mice, + denotes significant differences vs. CFA-injected mice treated with vehicle, # denotes significant differences vs. CFA-injected mice treated with CORM-2, and $ denotes significant differences vs. CFA-injected mice treated with CoPP (*p* < 0.05, one-way ANOVA, Student–Newman–Keuls test). Examples of western blots for HO-1 (32 kDa), NOS1 (160 kDa), NOS2 (100 kDa), CD11b/c (160 kDa), GFAP (50 kDa), GAPDH (37 kDa), p-ERK 1/2/total ERK 1/2 (42–44 kDa), and p-JNK/total JNK (46–54 kDA) are shown (**E**). The levels of phosphorylated proteins are indicated relative to the corresponding total protein levels, while the levels of the remaining proteins are relative to those of GAPDH. Results are expressed as mean ± SEM; *n* = 4 samples per group.

Our data showed that CoPP and CORM-2 treatments augmented the expression of HO-1 in the LC of CFA-injected WT mice (*p* < 0.001; one-way ANOVA vs. naïve and CFA-injected mice treated with vehicle) (Figure 2A), and the enhanced expression of NOS1 (Figure 2B) and GFAP (Figure 2F) caused by peripheral inflammation (*p* < 0.024; one-way ANOVA compared to the corresponding naïve vehicle-treated animals) was normalized by both CoPP and CORM-2 treatments. Moreover, the enhanced expression of p-ERK 1/2 caused by peripheral inflammation (*p* < 0.005; one-way ANOVA vs. naïve vehicle-treated mice) was normalized by CoPP treatment (Figure 2G). None of these treatments changed the unaltered expression of NOS2 (Figure 2C), CD11b/c (Figure 2D), or p-JNK (Figure 2H) in the LC of CFA-injected mice.

### 2.3. Effects of CoPP and CORM-2 on HO-1, NOS2, CD11b/c, GFAP, p-ERK 1/2, and p-JNK Expression in the LC of NOS1-KO Mice with Peripheral Inflammation

**Figure 3:**
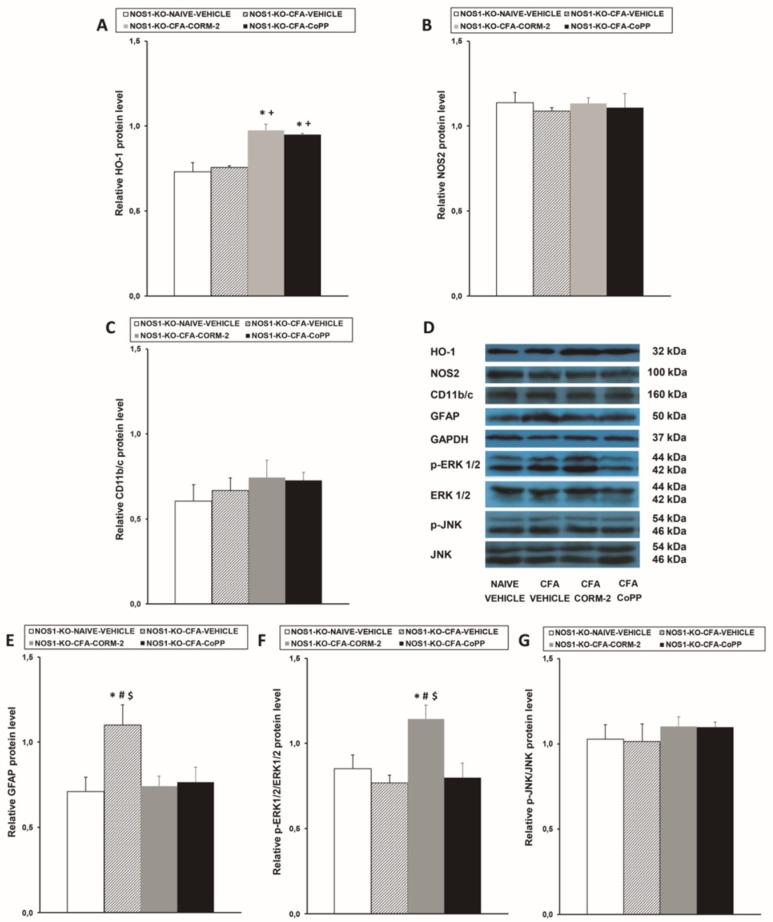
Effects of CoPP and CORM-2 on the protein levels of HO-1, NOS2, CD11b/c, GFAP, p-ERK 1/2, and p-JNK in the LC of NOS1-KO mice. Protein levels of HO-1 (**A**), NOS2 (**B**), CD11b/c (**C**), GFAP (**E**), p-ERK 1/2 (**F**), and p-JNK (**G**) in the LC of CFA-injected NOS1-KO mice treated with vehicle, CORM-2, or CoPP are represented. These levels from naïve mice treated with vehicle are also represented as controls. In all panels, * represents significant differences vs. naïve vehicle-treated mice, + represents significant differences vs. CFA-injected mice treated with vehicle, # represents significant differences vs. CFA-injected mice treated with CORM-2, and $ represents significant differences vs. CFA-injected mice treated with CoPP (*p* < 0.05, one-way ANOVA, Student–Newman–Keuls test). Examples of western blots for HO-1(32 kDa), NOS2 (100 kDa), CD11b/c (160 kDa), GFAP (50 kDa), GAPDH (37 kDa), p-ERK 1/2/total ERK 1/2 (42–44 kDa), and p-JNK/total JNK (46–54 kDA) are shown (**D**). The levels of phosphorylated proteins are relative to the total levels of the corresponding proteins, while the levels of the remaining proteins are relative to those of GAPDH. Results are expressed as mean ± SEM; *n* = 4 samples per group.

Similar to what observed in WT mice, while peripheral inflammation did not modify the protein levels of HO-1 in the LC of NOS1-KO animals (Figure 3A), HO-1 expression was significantly augmented in CoPP- and CORM-2-treated animals (*p* < 0.001; one-way ANOVA vs. naïve and CFA-injected mice treated with vehicle). None of these treatments altered the expression of NOS2 (Figure 3B), CD11b/c (Figure 3C), and p-JNK (Figure 3G) in the LC of CFA-injected NOS1-KO mice and, although peripheral inflammation did not activate ERK 1/2, an increased expression of p-ERK 1/2 was detected in NOS1-KO mice treated with CORM-2 (*p* < 0.019 vs. naïve and CFA-injected mice treated with vehicle or CoPP) (Figure 3F). In addition, the overexpression of GFAP induced by CFA was completely inhibited by CORM-2 and CoPP treatments (Figure 3E). 

### 2.4. Effects of CoPP and CORM-2 on HO-1, NOS1, CD11b/c, GFAP, p-ERK 1/2, and p-JNK Expression in the LC from NOS2-KO Mice with Peripheral Inflammation

**Figure 4:**
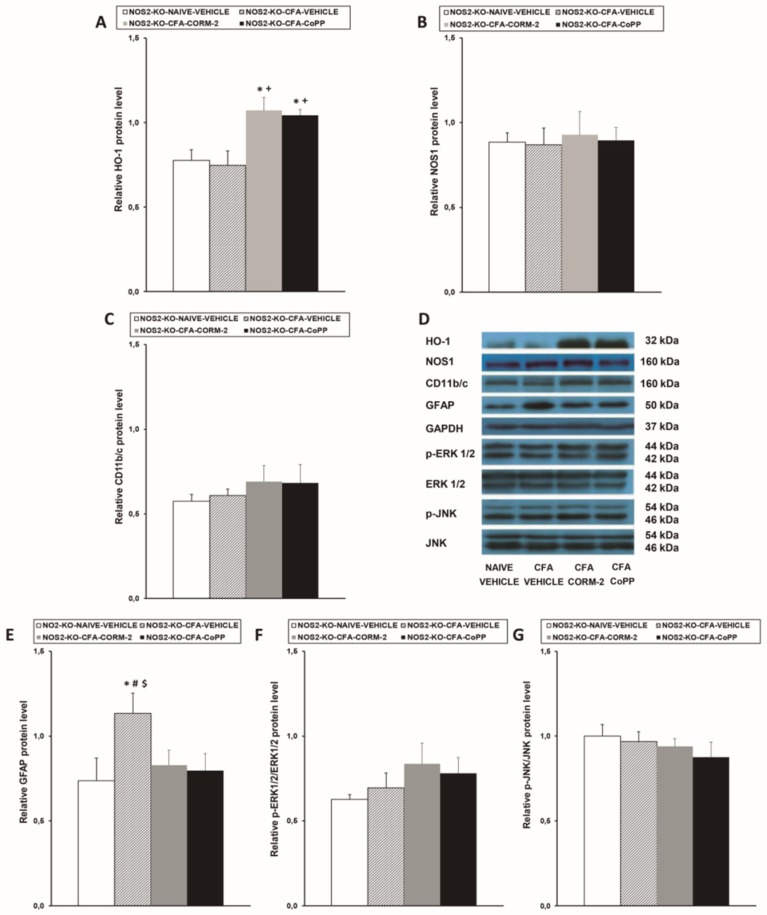
Effects of CoPP and CORM-2 on the protein levels of HO-1, NOS1, CD11b/c, GFAP, p-ERK 1/2, and p-JNK in the LC of NOS2-KO mice. Protein levels of HO-1 (**A**), NOS1 (**B**), CD11b/c (**C**), GFAP (**E**), p-ERK 1/2 (**F**), and p-JNK (**G**) in the LC of CFA-injected NOS2-KO mice treated with vehicle, CORM-2, or CoPP are represented. These levels from naïve mice treated with vehicle are also represented as controls. In all panels, * denotes significant differences vs. naïve vehicle-treated mice, + represents significant differences vs. CFA-injected mice treated with vehicle, # represents significant differences vs. CFA-injected mice treated with CORM-2, and $ represents significant differences vs. CFA-injected mice treated with CoPP (*p* < 0.05, one-way ANOVA, Student–Newman–Keuls test). Examples of western blots for HO-1 (32 kDa), NOS1 (160 kDa), CD11b/c (160 kDa), GFAP (50 kDa), GAPDH (37 kDa), p-ERK 1/2/total ERK 1/2 (42–44 kDa), and p-JNK/total JNK (46–54 kDA) are shown (**D**). The levels of phosphorylated proteins are relative to the total levels of the corresponding proteins, while the levels of the remaining proteins are relative to those of GAPDH. Each column represents the mean, and the vertical bars indicate the SEM; *n* = 4 samples per group.

Similar to what observed in NOS1-KO mice, although peripheral inflammation did not change the expression of HO-1 in the LC of NOS2-KO mice (Figure 4A), CoPP and CORM-2 treatments significantly enhanced its expression (*p* < 0.007; one-way ANOVA vs. naïve and CFA-injected mice treated with vehicle). Moreover, repeated treatment with CORM-2 or CoPP significantly reduced the enhanced expression of GFAP induced by peripheral inflammation in the LC (Figure 4E). In contrast, neither CORM-2 nor CoPP treatments altered the unchanged levels of NOS1 (Figure 4B), CD11b/c (Figure 4C), p-ERK 1/2 (Figure 4F), or p-JNK (Figure 4G) in the LC of CFA-injected NOS2-KO mice.

## 3. Discussion

This study revealed that the systemic repeated administration of CoPP differentially inhibited allodynia and hyperalgesia caused by CFA in WT and NOS1-KO or NOS2-KO mice. Moreover, CoPP and CORM-2 treatments induced HO-1 overexpression and inhibited activated astroglia in the LC of all genotypes. Both treatments also normalized the upregulation of NOS1 caused by peripheral inflammation in WT mice. Moreover, peripheral inflammation activated ERK 1/2 in the LC of WT animals but not in NOS1-KO or NOS2-KO mice, and only CoPP treatment inhibited ERK 1/2 phosphorylation. 

Our findings indicated that treatment with CoPP throughout 11 consecutive days inhibited the mechanical and thermal hypersensitivity triggered by peripheral inflammation in a different time-dependent manner in WT and NOS1- or NOS2-deficient mice. That is, the antiallodynic and antihyperalgesic effects of CoPP in WT mice were stronger than in NOS1-KO or NOS2-KO mice after 4 to 7 days of treatment. These data demonstrate the involvement of both NOS isoforms in the analgesic effects of CoPP during inflammatory pain and reveal an interaction between HO-1 and NOS1/NOS2 isoenzymes in chronic inflammatory pain conditions. Our results are in agreement with the analgesic effects induced by HO-1 inducers during acute [33] and neuropathic pain [2,4,21,34,35]. Our findings also support the improved antinociceptive actions of carbon monoxide, liberated by CORM-2, in WT versus both KO mice with inflammatory pain [20] and further demonstrate the crucial role played by HO-1 in the interaction between carbon monoxide and nitric oxide during the management of inflammatory pain.

It is well known that LC is a supraspinal structure implicated in the control of pain, but the effects induced by chronic treatment with CoPP or CORM-2 during inflammatory pain in this brain area had not been investigated previously. In previous works, we showed that the repetitive administration of CoPP or CORM-2 upregulated HO-1 levels in paws and dorsal root ganglia of animals with inflammatory pain [20,36] as well as in spinal cords and sciatic nerves from diabetic mice [4,35]. Recently, an augmented expression of HO-1 was also demonstrated in the prefrontal cortex and hippocampus of WT mice with neuropathic pain repeatedly treated with CORM-2 or CoPP [22]. In this study, we demonstrated for the first time that, under inflammatory pain circumstances, both HO-1 and carbon monoxide inducers improved HO-1 levels in the LC of WT, NOS1-KO, and NOS2-KO mice. These data agree with those reporting augmented expression of HO-1 induced by CoPP and CORM-2 treatments in the central nervous system of WT mice with neuropathic pain [22] and further support data showing the upregulation of this enzyme in the paw of CFA-injected NOS1- and NOS2-deficient mice systemically treated with CORM-2 [20]. Our results reveal the central antioxidant effects induced by these treatments during inflammatory pain in the presence or absence of NOS enzymes and suggest that the enhanced expression of HO-1 produced by CoPP and CORM-2 in the LC might be involved in the antinociceptive effects of these compounds during inflammatory pain.

In this study, an increased expression of NOS1, but not of NOS2, was also revealed in the LC of WT mice with peripheral inflammation. This is in agreement with observations in inflamed paws, showing the relevant role performed by NOS1 in the maintenance of inflammatory pain in the central and peripheral nervous system [37,38]. Moreover, the fact that the systemic administration of CoPP and CORM-2 inhibited NOS1-upregulation in the LC revealed the central anti-inflammatory effects induced by these compounds during inflammatory pain. In contrast to NOS1, no changes in the protein levels of NOS2 were observed in the LC of these animals. These data are in agreement with the observed lack of changes in the expression of this enzyme in the dorsal root ganglia and spinal cords of WT mice at day 14 after CFA injection [30]. All of these data suggest that, in chronic peripheral inflammatory pain conditions, the effects induced by CoPP and CORM-2 treatments in the LC are mainly produced via regulating NOS1 expression.

The implication of spinal glia in the progress of chronic pain is well recognized, but less is known about its activation in supraspinal structures after peripheral inflammatory pain. Thus, in this study, we examined if peripheral inflammation induced astroglial and/or microglial activation in the LC 14 days after CFA injection. Our results showed that CFA induced astroglial activation in the LC of WT, NOS1-KO, and NOS2-KO mice. No changes in the expression of CD11b/c (a microglial marker) were detected in the LC of any genotype, confirming the crucial role of astroglia in the maintenance of chronic inflammatory pain [39,40]. Interestingly, CORM-2 and CoPP inhibited the overexpression of GFAP (an astroglial marker) in the LC of all genotypes, showing that the systemic administration of these compounds has anti-inflammatory properties in the LC. Our results also suggest the participation of astroglia in the analgesic effects of CORM-2 and CoPP during inflammatory pain. These findings are supported by the antinociceptive actions produced by the treatment with selective astroglial cell inhibitors, such as fluorocitrate and α-aminoadipate, during chronic pain [41,42].

The participation of MAPK in the development and maintenance of chronic pain is well documented [29,43]. Thus, under inflammatory pain conditions, the expression of ERK1/2 and JNK are activated in the spinal cord [30]. In this work, we analyzed the actions of CoPP and CORM-2 on the expression of these MAPK in the LC of WT and NOS-deficient mice. As occurs in other animal pain models [32,44], CFA-induced peripheral inflammation activated ERK 1/2 in the LC of WT mice. Curiously, this effect was not detected in NOS1-KO or NOS2-KO mice, suggesting that nitric oxide generated by these isoforms is involved in the plasticity changes induced by chronic peripheral inflammation in LC. Moreover, while CoPP normalized ERK 1/2 activation in WT animals, CORM-2 did not alter or enhanced p-ERK 1/2 levels in WT and NOS1-KO mice, respectively, thus revealing that ERK 1/2 activation induced by peripheral inflammation in the LC might be modulated by the systemic treatment with the antioxidant enzyme HO-1. The lack of effects of CORM-2 on the expression of p-ERK 1/2 in WT mice confirmed similar results obtained in the central and peripheral nervous system of animals with neuropathic pain [22,45], suggesting that CORM-2 might act via inhibiting other pathways implicated in the regulation of inflammatory pain. Finally, although more studies are required to explain this phenomenon, the increased expression of p-ERK 1/2 induced by CORM-2 in NOS1-KO mice is an indication of the plausible involvement of NOS1 in ERK 1/2 activation induced by CORM-2 in WT mice.

In conclusion, this study reveals an interaction between HO-1 and NOS1/NOS2 enzymes during peripheral inflammation and shows that CoPP treatment inhibited inflammatory pain by improving HO-1 expression and decreasing NOS1 overexpression, which would restrict the activation of astroglia and subsequent ERK 1/2 activation in the LC.

## 4. Materials and Methods 

### 4.1. Animals

Experiments were performed in male NOS1-KO (C57BL/6 J background) and NOS2-KO mice (C57BL/6 J background) acquired from Jackson Laboratories (Bar Harbor, ME, USA). WT mice with the same genetic background (C57BL/6J) were obtained from Envigo Laboratories (Barcelona, Spain). Animals weighing 21–25 g were housed under 12 h/12 h light/dark conditions and controlled temperature (22°C) and humidity (66%). Animals with free access to food and water were utilized after 7 days acclimatization to the housing conditions. Experiments were performed between 9:00 a.m. and 5:00 p.m., executed in accordance to the animals guidelines of the European Communities Council (86/609/ECC, 90/679/ECC; 98/81/CEE, 2003/65/EC, and Commission Recommendation 2007/526/EC), and approved by the Comitè d’Ètica en Experimentació Animal of Universitat Autònoma de Barcelona (number: 1325R5, 29 November 2013). Maximal efforts to minimize the quantity of mice employed and their suffering were made.

### 4.2. Chronic Inflammatory Pain Induction

Chronic inflammatory pain was provoked with the sub-plantar injection of CFA (30 μL) (Sigma-Aldrich, St. Louis, MO, USA) into the right hind paw under brief anesthetic conditions with isoflurane (3% induction, 2% maintenance) according to the method used by our group [38]. Mechanical allodynia and thermal hyperalgesia were assessed with the von Frey filaments and plantar tests, respectively. 

### 4.3. Nociceptive Behavioral Tests

Mechanical allodynia was estimated by determining the hind paw withdrawal response to von Frey filament stimulation. Thus, mice were positioned in methacrylate cylinders (20 cm high, 9 cm diameter) with a wire grid bottom through which the von Frey filaments (North Coast Medical, Inc., San Jose, CA, USA) were applied according to the up–down paradigm [46]. A filament of 0.4 g was applied first, and one of 3.5 g as a cut-off. The strength of the next filament was reduced or augmented depending on the response. Withdrawal, shaking, or licking the paw were considered nociceptive-like reactions.

Thermal hyperalgesia was assessed according to previous methods [47]. Paw withdrawal latency in reply to a radiant heat was assessed using the plantar test device (Ugo Basile, Italy). Mice were placed in methacrylate cylinders, 20 cm high × 9 cm diameter, situated on a glass surface. The heat source was situated under the plantar surface of the hind paw and activated with a light beam. A cut-off time of 12 s was utilized to avoid tissue damage. The mean paw withdrawal latencies were calculated from the average of 2–3 separate trials, taken at 5 min intervals to prevent thermal sensitization. 

In both tests, the animals were habituated to the environment for 1 h before the experiment. Both ipsilateral and contralateral paws were tested.

### 4.4. Western Blot Analysis

Mice were euthanized by cervical dislocation after 0 (naïve) or 14 days from CFA injection. LC were extracted and preserved at 80 °C until use. Samples from four animals were combined to have satisfactory proteins levels to analyze HO-1, NOS1, NOS2, CD11b/c, GFAP, pERK1/2/ERK1/2, and p-JNK/JNK in LC by western blot assay. The homogenization of tissues was made in cold lysis buffer (50 mM Tris·Base, 150 nM NaCl, 1% NP-40, 2 mM EDTA, 1mM phenylmethylsulfonyl fluoride, 0.5 Triton X-100, 0.1% sodium dodecyl sulfate, 1 mM Na3VO4, 25 mM NaF, 0.5% protease inhibitor cocktail, and 1% phosphatase inhibitor cocktail). All reagents were acquired from Sigma-Aldrich (St. Louis, MO, USA), except for NP-40 which was bought from Calbiochem (Darmstadt, Germany). After solubilization for 1 h at 4 °C, crude homogenates were sonicated for 10 s and centrifuged at 700× *g* for 15 min at 4 °C. The supernatant (60 µg of total protein) was mixed with 4X Laemmli loading buffer and loaded onto a 4% stacking/10% separating sodium dodecyl sulfate polyacrylamide gels. Proteins were electrophoretically transferred onto a polyvinylidene fluoride membrane for 120 min and successfully blocked with phosphate-buffered saline containing 5% nonfat dry milk or Tris-buffered saline with Tween 20 containing 5% bovine serum albumin for 75 min; they were then incubated with specific rabbit primary antibodies anti HO-1 (1:300; Abcam, Cambridge, United Kingdom), NOS1 (1:200; Abcam, Cambridge, United Kingdom), NOS2 (1:100; Abcam, Cambridge, United Kingdom), CD11b/c (1:160, Novus Biologic, Littleton, CO, USA), GFAP (1:3000, Novus Biologic, Littleton, CO, USA), phospho-ERK 1/2 and total ERK 1/2 (1:250; Cell Signaling Technology, Danvers, MA, USA), phospho-JNK and total JNK (1:250; Cell Signaling Technology, Danvers, MA, USA), or glyceraldehyde-3-phosphate dehydrogenase antibody (GAPDH) (1:5000, Merck, Billerica, MA, USA) overnight at 4 °C. The blots were then incubated with anti-rabbit secondary polyclonal antibodies conjugated to horseradish peroxidase (GE Healthcare, Little Chalfont, Buckinghamshire, UK) for 1 h at r.t. Proteins were detected by using chemiluminescence reagents provided in the ECL kit (GE, Healthcare, Little Chalfont, Buckinghamshire, UK) and exposure onto hyperfilms (GE, Healthcare, Little Chalfont, Buckinghamshire, UK). Blots’ intensity was quantified by densitometry.

### 4.5. Experimental Procedure

In WT, NOS1-KO, and NOS2-KO mice baseline responses were established in von Frey filaments and plantar tests. All mice were tested at days 3, 4, 7, 10, and 14 after CFA injection. 

The animals were intraperitoneally injected with vehicle, 2.5 mg/kg CoPP, or 5 mg/kg CORM-2, two times a day for 11 days, from day 4 to day 14 after CFA injection, according to earlier studies [2,20]. The antinociceptive effects of CoPP were evaluated at 1, 4, 7, and 11 days from its administration. The investigator who made these experiments was blinded to the treatments.

We assessed the effects of CoPP and CORM-2 on the expression of HO-1, NOS1, NOS2, CD11b/c, GFAP, pERK 1/2, ERK 1/2, p-JNK, and JNK in the LC of WT, NOS1-KO, or NOS2-KO mice 14 days after CFA injection by western blot assay. For each genotype, naïve mice treated with vehicle were used as controls (*n* = 4 samples per group).

### 4.6. Drugs

CoPP was purchased from Frontier scientific (Livchem GmbH & Co., Frankfurt, Germany), and CORM-2 from Sigma-Aldrich (St. Louis, MO, USA). Both compounds were dissolved in dimethyl sulfoxide (DMSO; 1% solution in saline), freshly prepared before use, and injected intraperitoneally at 10 mL/kg of body weight, 3 h before testing, twice a day. Control animals received the same volume of vehicle.

### 4.7. Statistical Analysis

The SPSS (version 17 for Windows, IBM, Madrid, Spain) was used to perform the statistical analysis. Data were expressed as mean ± standard error of the mean (SEM). For each behavioral test and time evaluated, the antiallodynic and antihyperalgesic effects of CoPP were evaluated by using a three-way analysis of variance (ANOVA) (genotype, treatment, and paw as factors of variation) followed by the pertinent one-way ANOVA and Student–Newman–Keuls test whenever required. For each genotype, alterations in the protein levels of HO-1, NOS1, NOS2, CD11b/c, GFAP, p-ERK 1/2, ERK 1/2, p-JNK, and JNK in the LC of CFA-injected mice treated with CORM-2, CoPP, or vehicle vs. naïve vehicle-treated mice were analyzed by a one-way ANOVA followed by the Student–Newman–Keuls test. A *p* < 0.05 was considered significant. 

## Abbreviations

HO-1	Heme oxygenase 1
LC	Locus coeruleus
WT	Wild-type
NOS1	Neuronal nitric oxide synthase
NOS2	Inducible nitric oxide synthase
KO	Knockout
CFA	Complete Freund’s adjuvant
CoPP	Cobalt protoporphyrin IX
CORM-2	Tricarbonyldichlororuthenium(II) dimer
ERK 1/2	Extracellular signal-regulated kinase 1/2
JNK	c-Jun N-terminal kinase
GAPDH	Glyceraldehyde-3-phosphate dehydrogenase
GFAP	Glial fibrillary acidic protein
SEM	Standard error of the mean
ANOVA	Analysis of variance
